# Medical Expert Evidence.—III

**Published:** 1909-06-12

**Authors:** 


### Medico-Legal Points.

### MEDICAL EXPERT EVIDENCE.—III.

Medical men may testify as to the probable result of known injuries upon the health and life of the person injured, and as to the probable duration of injuries, and as to the probability that the injury will cause future pain and suffering, as well as the effect of the injury upon the future ability to work or attend to other affairs of life of the person injured, and as to ultimate curability, or probable recovery, or permanency of the effects of the injury, and as to the liability that the injured person will be subject to other dangers resulting from the injury; such opinions are not objectionable as speculative, since that which happens in the natural and ordinary course of events may be assumed to happen with reasonable certainty. Likewise the question whether a described accident was capable of producing certain physical results is a proper one for a medical expert; and a physician may give his opinion as to the probability of the recurrence of a condition resulting from an injury, and as to the probable necessity for future medical attendance. The opinions of medical men as to the location, character, and probable consequences of wounds are competent evidence in a prosecution for homicide, or assault with intent to kill. And this is the rule whether based upon observation or upon a description of the wounds by other witnesses. And a surgeon is competent to express an opinion as to whether or not a wound is a mortal one, and as to the nature of the instrument which would produce such a wound, and as to whether a wound was inflicted before or after death; and he may give his opinion as to the probable cause of a wound, and as to the amount of force necessary to cause it. The cause of the particular wound in question, however, is a question for the jury, and not one upon which an expert may express an opinion. And so is the question whether a wound was accidentally or purposely inflicted.

A duly qualified medical expert, however, who examined the body of a deceased person shortly after the infliction of a wound causing his death, may give an opinion as to whether it was produced by a near shot or one fired from a distance. And a surgeon of experience who had seen many gunshot powder marks and powder burns, and knew of his own knowledge the distance between the discharged weapons and the bodies fired upon, is sufficiently qualified to give an opinion as to the distance at which a certain pistol would produce powder marks on the skin; though the rule would be different if the witness did not appear to be thus properly qualified as an expert on powder marks. A physician making a post-mortem examination of a person killed by a blow upon his head is competent to give an opinion as to the direction from which the blow was delivered. And a medical expert may give his opinion as to the range, after entering the body, of a shot which caused death, taking into consideration the bone, muscle, and other substances through which it had to pass.

Stains of blood found upon the person or clothing of a deceased person are recognised as an ordinary indication of homicide, and are competent evidence of its commission even in the absence of proof that the stains were in fact blood-stains, constituting primary and legitimate and not secondary evidence of homicide, its weight being a question for the jury. And the witness need not be a chemist or physician or expert to enable him to testify that certain spots or stains were in fact blood-spots or blood-stains; and the inferences as to the relative positions of two combatants based upon the appearances of blood-stains are within the domain of common experience, and not a matter of science. In a case resting upon circumstantial evidence, however, proof of apparent blood-spots, without chemical analysis, does not warrant the legal presumption that the substance was blood, because of the similarity of stains made by other substances; and where an effort is made to distinguish between human blood and that of some animal, the question is one of science, requiring the application of great skill and knowledge, upon which the testimony must be that of an expert.

A medical examination and chemical analysis are more important in cases of alleged poisoning than symptoms; and proof of symptoms of poisoning—especially when unsatisfactory and unreliable—will not warrant conviction for poisoning in the absence of chemical analysis and application to the stomach and its contents of approved tests for the discovery of poison. And medical men may testify, in a prosecution for poisoning, to a chemical analysis made by them of the stomach of the deceased, and to the tests applied for detecting the existence of poison, though they are not professional chemists, and have no experience in the analysis of poison; but a chemical analysis for the purpose of discovering poison would be of less weight if conducted by persons without practical experience than if conducted by practical chemists whose conclusions were based upon experience as well as upon study. The question of the condition of the stomach of a person alleged to have been poisoned is likewise one involving skill and science, and a proper subject for expert testimony. But specialists of experience are alone competent to testify as to the effect of particular drugs upon the human system, though medical experts may properly testify as to the effect of poisons from information derived from the writings of standard authors on the subject. And one who had made a chemical analysis of the stomach of a person alleged to have been poisoned may testify as an expert concerning the effect of strychnine upon the human system when he is a chemist and toxicologist, though not a physician and surgeon.

A medical expert who has examined or treated a person claiming to be ill or injured is competent to give an opinion from the general appearance, actions and looks of the patient, and from his examination and statements as to whether or not his trouble or injury was imaginary, feigned, or real. A physician is not competent, however, to give his opinion that a plaintiff in an action for personal injury is shamming before the jury, a physician being no better qualified on that subject than the jurors. And a medical expert cannot give his opinion on the question whether or not a person claiming injury or illness is a malingerer. And an opinion that a person was simulating pain or suffering is incompetent, when based upon personal acquaintance with such person, or some other reason not within the range of expert testimony.

The jury is the judge of the weight to be attached to the opinions of medical experts. Jurors are not controlled by medical opinions; the medical expert cannot be put in the place of the jury and allowed to decide the case. Such opinions are to be considered in connection with the other evidence, and given just weight; but the jury must determine for itself, from the whole evidence, the question at issue, and their value must be made to depend upon the agreement or non-agreement of the facts assumed as their basis with the actual facts of the particular case and upon the opportunities of the witness to acquire skill and knowledge and the use he makes of these opportunities.

When, however, the case concerns a highly specialised branch of the medical art, with respect to which a layman could have no knowledge, the court must be dependent upon expert testimony; and in such cases, in the absence of such evidence, it is improper to submit the case to the jury. And evidence of medical and scientific persons, physicians, surgeons, and chemists, by whom a body had been inspected and examined either at the time of its discovery or shortly after, and their opinions with reference to it are competent and of great value in a prosecution for homicide, in establishing the *corpus delicti*. But such opinions can only be regarded as scientific, so as to be entitled to additional weight so far as they relate to physical man, and his diseases, and their means of cure. And where a medical opinion is given by a physician, it becomes a proper subject for cross-examination for the purpose of ascertaining his qualifications and fairness and impartiality, and the consequent weight to which his opinion is entitled; and for this purpose he may be asked as to his experience and reading in similar cases, and as to his treatment of the patient and the nature of the case in hand. Nor is it improper on cross-examination to ask physicians who attended an injured person by whom they were sent and paid. And it may be shown that a medical expert charged or expected to receive greater compensation than the fees allowed by law, and that he is in the employ of one of the litigants regularly or frequently as an expert witness.

The object of calling an expert into court being to obtain a personal opinion from him, together with the reasons therefor, it has become a rule of very general adoption not to admit professional books in evidence, nor even to allow the expert to quote their opinions by substitution for his own. Yet he may be asked the ground of his judgment and opinion, which might in some degree be founded on these books, for it could be easily shown in any case that no man had ever framed an opinion without elementary assistance from some objective source, and as books are the chief instructors of educated men, while experience is only the practical application of their teachings to the necessities of professional life, it follows that some book knowledge is, in fact, at the bottom of all opinions. The expert, of course, may consult them as much as he pleases before going into court, and may even refer to them as representing the accepted opinions of the profession, but he cannot read from them. The reason of this rule is founded on the principle that the expert is called to express a personal opinion upon a state of facts of variable interpretation, and if a book could pronounce it as well it would be superfluous to call him. He is summoned for the purpose of giving aid, through the employment of his professional experience, to the administration of justice, and when he undertakes to read from books a conclusion which he claims to adopt as his judgment in the premises, he certainly does to that extent attempt to substitute another's opinion for his own. Yet he may, after stating his opinion, give the reasons for it, as founded upon the concurrent observation and experience of others recorded in their works, and thus contributing to furnish him with that general knowledge, by means of which he is confirmed in his competency to act as a skilled witness. Accordingly it has been held that medical witnesses, in giving their opinions as experts, are not confined to the results of their own observation and experience, but may give opinions based upon information derived from books. But the naked statements of books of science, not verified by his own experience, are of no more authority than the books themselves, and the opinions given in such books are not legal evidence.

